# Miscellanea

**Published:** 1855-11

**Authors:** 


### MISCELLANEA.

*Another Change.*—The two medical journals of Virginia, the Virginia Medical and Surgical Journal and Stethescope, will be united on the first of January next, under the style of the Virginia Medical Journal, with Drs. McCaw, Peebles & Otis as editors.

*Western Enterprise.*—Renouard's History of Medicine, a volume of eight hundred pages, octavo, translated by Prof. Comegys, of the Miami Medical College, has been recently published in beautiful style by a Cincinnati house. A translation of this same work, prepared with much care, a few years since, by a distinguished physician of New York, was offered to the medical publishers of that city without finding one willing to undertake its issue.

